# Deterioration of Organ Function As a Hallmark in Sepsis: The Cellular Perspective

**DOI:** 10.3389/fimmu.2018.01460

**Published:** 2018-06-26

**Authors:** Michael Bauer, Sina M. Coldewey, Margit Leitner, Bettina Löffler, Sebastian Weis, Reinhard Wetzker

**Affiliations:** ^1^Department of Anesthesiology and Intensive Care Medicine, Jena University Hospital, Jena, Germany; ^2^Center for Sepsis Control and Care (CSCC), Jena University Hospital, Jena, Germany; ^3^Septomics Research Center, Jena University Hospital, Jena, Germany; ^4^Institute of Medical Microbiology, Jena University Hospital, Jena, Germany; ^5^Center for Infectious Disease and Infection Control, Jena University Hospital, Jena, Germany

**Keywords:** sepsis, organ dysfunction, metabolic adaptation, disease tolerance, signaling

## Abstract

Development of organ dysfunction discriminates sepsis from uncomplicated infection. The paradigm shift implicated by the new sepsis-3 definition holds that initial impairment of any organ can pave the way for multiple organ dysfunction and death. Moreover, the role of the systemic inflammatory response, central element in previous sepsis definitions, has been questioned. Most strikingly, a so far largely underestimated defense mechanism of the host, i.e., “disease tolerance,” which aims at maintaining host vitality without reducing pathogen load, has gained increasing attention. Here, we summarize evidence that a dysregulation of critical cellular signaling events, also in non-immune cells, might provide a conceptual framework for sepsis-induced dysfunction of parenchymal organs in the absence of significant cell death. We suggest that key signaling mediators, such as phosphoinositide 3-kinase, mechanistic target of rapamycin, and AMP-activated protein kinase, control the balance of damage and repair processes and thus determine the fate of affected organs and ultimately the host. Therapeutic targeting of these multifunctional signaling mediators requires cell-, tissue-, or organ-specific approaches. These novel strategies might allow stopping the domino-like damage to further organ systems and offer alternatives beyond the currently available strictly supportive therapeutic options.

## Introduction

The advent of the new sepsis-3 definition, published in 2016 prompted a reappraisal of organ dysfunction as the hallmark of sepsis. Sepsis is now defined as “life-threatening organ dysfunction caused by a dysregulated host response to infection” (1). With this definition, the conceptual focus shifted from exclusive attention to the host inflammatory response, i.e., Systemic Inflammatory Response Syndrome (2, 3), to the multifactorial tissue damage occurring during the progression of infection to sepsis. In the current conceptual framework, sepsis can be construed as a pathogen-induced imbalance of host damage and repair processes that trigger failure of either *resistance* or “*disease tolerance*” mechanisms (4). In these processes, metabolic adaptation is of outstanding significance (1, 5–7).

Certain critical mediators, most notably related to phosphoinositide 3-kinase (PI3K), mechanistic target of rapamycin (mTOR), and AMP-activated protein kinase (AMPK) signaling, have dual functions in metabolism and defense signaling. In this “perspective,” we summarize evidence that substantial changes in these signaling pathways during sepsis occur not only in the immune system but also in parenchymal cells. To target (parenchymal) cellular metabolic and signaling dysfunctions will open new avenues for therapeutic interventions.

## Septic Shock, Sepsis-Related Dysoxia, and Energy Crisis

**Figure 1:**
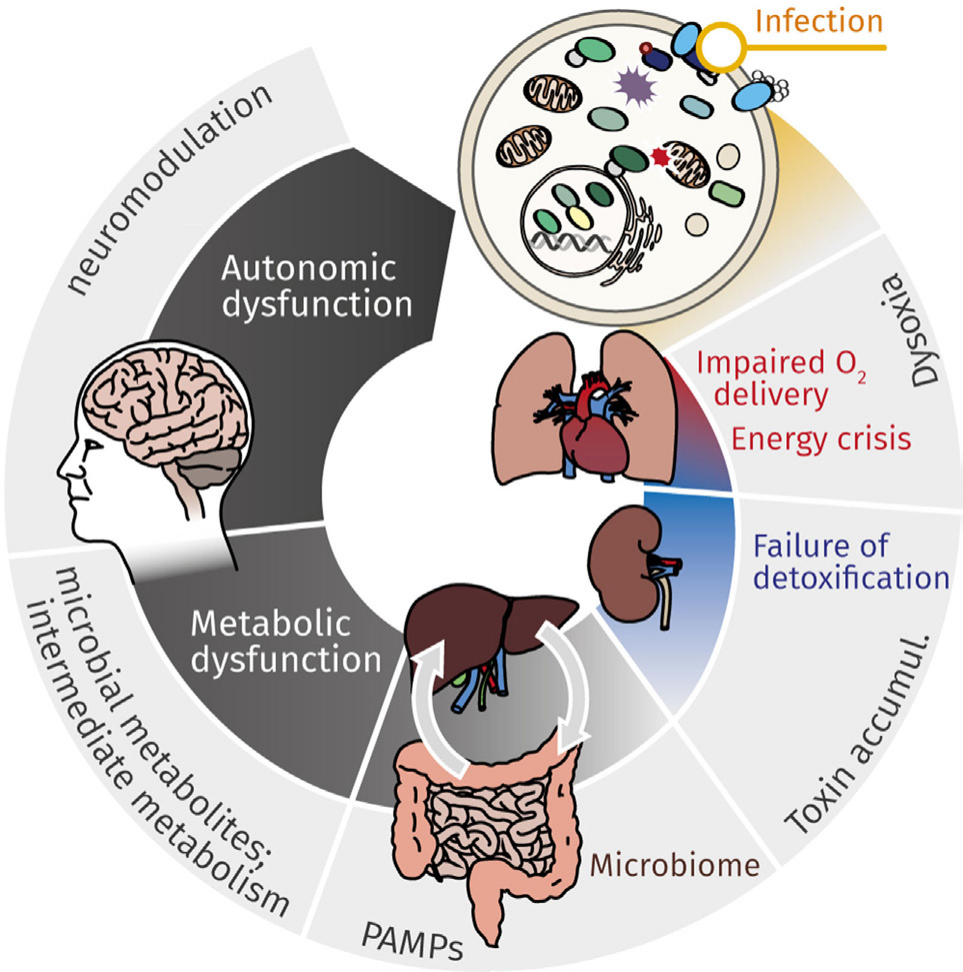
Mutual effects of cellular energy metabolism and organ dysfunction cause continuous deterioration during sepsis. While cardiovascular impairment leads to reduced O_2_ delivery, metabolic dysfunction, most markedly mitochondrial dysfunction, contributes to impaired cellular energy supply. This energy crisis contributes to failure of detoxification, which in turn affects primarily kidney and liver. Accumulating toxins, gut microbiome with (antibiotic-induced) dysbiosis, and microbial metabolites (along the gut–liver axis, *via* organ–organ cross-talk) aggravate and perpetuate deteriorating organ function. The resulting metabolic catastrophe ultimately results in neurologic manifestations and autonomic dysfunction, which again contributes to continuing the downward spiral of metabolic derangement and organ dysfunction.

Severe metabolic dysregulation and circulatory failure are central to the definition of septic shock (1, 3) and sepsis mortality rises dramatically when there is cardiovascular impairment (8, 9). Microvascular thrombosis and low blood pressure act together with the sepsis-induced endothelial capillary leakage syndrome. These factors can aggravate and perpetuate organ damage through impaired oxygen availability (10). In addition, impaired mitochondrial function and biogenesis as well as presumably mitophagy have been identified as common features in non-survivors of sepsis (11). These findings support our hypothesis that impaired cellular metabolic processes occur early and widespread across the organism during sepsis development. We assume that the resulting “metabolic crisis” in multiple organs is accompanied by an increasing “misfiring” of signaling mediators, further deteriorating metabolic functions. Predominantly key players of anabolic processes, such as PI3K or mTOR, or of cellular maintenance, including AMPK, get out of control (12, 13). These cellular metabolic dysfunctions also impact detoxification mechanisms, the failure of which perpetuates metabolic deterioration (see Figure 1). Accordingly, “septic shock” is now defined as a “subset of sepsis in which underlying circulatory and cellular/metabolic abnormalities are profound enough to substantially increase mortality” (1). Thus, the current concept of (multi-) organ dysfunction focuses on the deleterious impact of already failed organ functions, most notably cardiovascular dysfunction, as a perpetuator of damage. Supposedly, interfering with the discussed signaling events will allow intervening at a much earlier stage.

## End Organ Failure—Insights from Systems Medicine

A paradigmatic example of sepsis-related organ failure is excretory dysfunction of the liver. Sepsis accounts for approximately 20% of admissions for jaundice, a rate that is only surpassed by malignant compression of the bile duct (14). Whereas the traditional view of liver failure would imply (pericentral) necrosis or apoptosis of parenchymal cells, this has been excluded as the predominant mechanism in septic shock by the pioneering work of Hotchkiss et al. (15) revealing that severe organ dysfunction occurs despite remarkably well preserved tissue structure. A systems biology approach analyzing the hepatocyte response to infection found reprogramming of metabolic functions in parallel with severity-dependent disruption of phase I and II biotransformation and excretory failure (16, 17). This characteristic phenotype of excretory failure critically depends on PI3Kγ signaling as a triggering event. This important role of PI3Kγ is also supported by interventions into upstream localized G protein-coupled receptors. As such, the long known role of C5aR in sepsis (18, 19), which signals through PI3Kγ, might reflect the central importance of the PI3K-Akt-mTOR axis. Taken together, these findings indicate that liver dysfunction does not result from a loss of viable parenchyma but from cellular dysfunctions that are potentially reversible and thus amenable to therapeutic intervention. Whereas inhibition of PI3Kγ could be a strategy to counter liver failure, the same signaling molecule might confer protection in other organs, e.g., in the heart (20). This highlights the need to achieve organ and cell-specific targeting of these processes in multi-organ dysfunction (21, 22).

## Altered Signaling in Sequential Stress Events and by Comorbidities

Liver dysfunction also strongly challenges overall metabolic and immunological homeostasis in the critically ill and frequently promotes progression to multi-organ failure. Prolonged hepatic dysfunction interferes with the adaptive immune system (23). Accordingly, pre-existing liver disease is a risk factor for the progression of bacterial infections to sepsis with increased odds ratios for hospitalization, ICU admission, and death (24). Similarly, patients with septic complications in the presence of chronic liver disease, most notably cirrhosis, have a poor prognosis due to development of acute-on-chronic liver failure (25). Altered signaling in chronic liver disease is closely connected to signaling processes mediating liver dysfunction during sepsis-triggered hepatic and extrahepatic organ failure (26, 27). In both conditions, metabolic master regulators, such as mTOR and AMPK, are in the center of the regulatory cascade. These and other key regulators, like the classical NFκB signaling pathway (28), probably govern the processes promoting damage and functional recovery. The underlying mechanisms might be amenable to therapy (1, 6, 7). Shock and cell death can further aggravate organ dysfunction, but in a small proportion of patients only (29).

## Imbalance Between Damage and Repair Processes as a Unifying Concept of Organ Failure

Organisms can counter infections *via* two distinct strategies: resistance and disease tolerance (4, 30–32). While resistance mechanisms and immunopathology have been in the focus of sepsis research for the past decades, disease tolerance and resilience remained largely underrated in animal and human biology (4).

Resistance mechanisms aim at reducing pathogen burden by pathogen destruction and elimination. The term disease tolerance summarizes a diversity of mechanisms that enable an organism to cope with a given stressor without eliminating it. In the context of infections and sepsis, these mechanisms provide tissue damage control and repair to sustain host tissue integrity and organ function even without reducing pathogen burden (32, 33). Disease tolerance can thus result in a relative increase in host fitness in the presence of a disease burden that would otherwise greatly reduce vitality (34). A disadvantage of these mechanisms is that inadequate tolerance can result in persistent infections and possibly relapses. Disease tolerance undoubtedly has a genetic basis but, as yet, this aspect has scarcely been investigated. Studies in *Drosophila* suggest that components other than the immune system are crucial for tolerance and endurance of infections (35).

Although we are far from knowing all factors involved, disease outcome seems to strongly depend on a fine balance between resistance and disease tolerance mechanisms that are further influenced by environmental factors [see, e.g., Ref. (36)].

## Therapeutic Strategies Derived from Stress Biology

That tolerance to infections is not exclusively linked to the immune system was further substantiated by the finding that anthracyclines, long known as chemotherapeutic agents, attenuated lung injury in a rodent model of sepsis (37). The protective effect can presumably be attributed to the induction of damage repair pathways *via* DNA damage responses (37). The underlying principle, systemic induction of maintenance and repair processes by stressors, such as cytotoxic agents or radiation, seems to be a general phenomenon, as demonstrated in *C. elegans* (38), among other organisms. These results indicate that tolerance can be pharmaceutically enhanced and that the principle of hormesis—a general fitness benefit of low doses of a stressor—is applicable to counter infections and sepsis (39).

Over the past century, however, the predominant medical interventions against infectious diseases have involved targeting resistance mechanisms, e.g., via vaccination, and direct reduction of pathogen load *via* antimicrobial drugs (40). But this strategy comes at the expense of drug resistance against many classes of antibiotics (41). Moreover, whereas this strategy has proven to be very successful against a broad range of infectious diseases, it has often failed in the treatment of sepsis (42). Futile antibiotic therapy in sepsis is a common phenomenon, even if the pathogen is tested sensitive to the given drug. This underlines the need for host-directed supportive therapies. In this connection, disease tolerance mechanisms emerge as therapeutic targets in the treatment of infectious diseases and offer fundamentally novel concepts in the treatment of sepsis (4, 32, 34, 43). Exploiting disease tolerance to counter sepsis also seems promising in view of increasing antibiotic resistance, as it does not generate selective pressure and cannot be opposed by antimicrobial resistance development. Thus, tolerance strategies are considered more stable (30, 44). Moreover, as tissue damage triggered by an excessive immune response is suggested to be the predominant cause of multi-organ failure, which results in poor disease outcome, damage control and repair mechanisms are promising targets for new therapeutic interventions. And, indeed, stress responsive genes that prevent the deleterious effects of damage-associated molecular patterns, such as free heme/iron (7, 45), DNA damage (37), or oxidative damage (46) have been shown in experimental systems to protect against bacterial infections without reducing host pathogen burden. In these tolerance settings, stabilizing organ functions has priority and pathogen eradication might be insufficient. However, unrestricted tolerance carries the risk of creating persistent reservoirs of pathogens in the host population (44, 47–49). To reduce this risk, therapeutically used tolerance responses have to be strictly controlled. Timing and extent of tolerance induction seem of eminent importance. Pathogen persistence and dissemination might be prevented by suppressing tolerance mediators or by provoking specific immune responses. Finally, pathogen persistence mechanisms might be affected directly to clear an infection and re-achieve balanced tolerance and resistance.

## Intracellular “Wiring” of Metabolism and Host Defense Mechanisms

**Figure 2:**
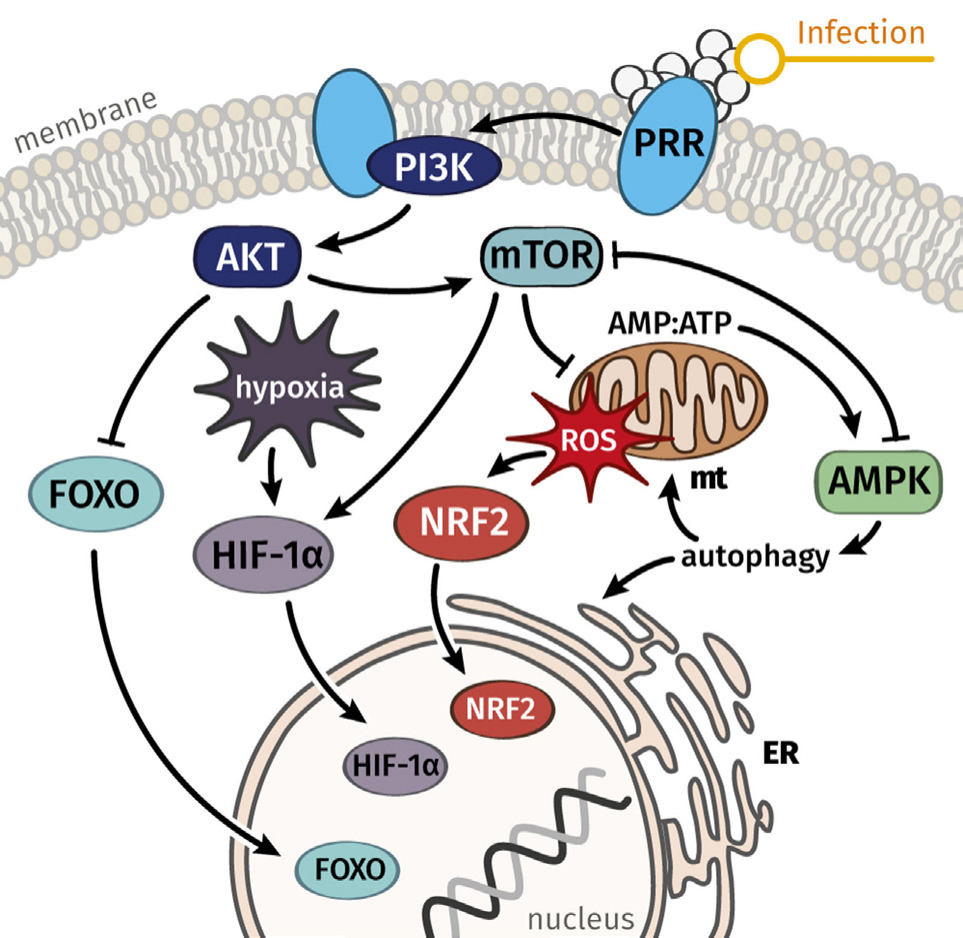
Key signal mediators connect stress responses to metabolism and can serve to explain a balance between damage and repair processes in immune cells and the majority of parenchymal cells. We hypothesize that dysregulated signaling and metabolic functions, which govern resistance responses and disease tolerance, are the common underlying cause of septic organ failure. Whereas AMPK is considered a key mediator of disease tolerance, mTOR and connected pathways regulate resistance responses and the metabolic reprogramming connected to immune activation. Abbreviations: AMPK, AMP-activated protein kinase; ER, endoplasmic reticulum, FOXO, forkhead box O; HIF-1α, hypoxia-inducible factor-1α; mt, mitochondrion; mTOR, mechanistic target of rapamycin; NRF2, nuclear factor erythroid 2-related factor 2; PI3K, phosphoinositide 3-kinase; PRR, pattern recognition receptor; ROS, reactive oxygen species.

Immunity and cellular metabolism are intricately connected, as the proteins PI3K, Akt, mTOR, HIF1alpha, and PKC are not only well described mediators of resistance responses but also drive energy-demanding anabolic processes, including glucose storage, protein synthesis, and proliferation. In contrast, disease tolerance is closely connected to cellular maintenance reactions, including the unfolded protein response and autophagy. Both processes are predominantly controlled by the signaling mediator AMPK. The interplay of mTOR and AMPK can, thus, serve as paradigm for the critical balance between resistance and disease tolerance. Apparently, both defense mechanisms become dysregulated during sepsis. Whereas excessive activation of resistance responses can lead to immunopathology, inappropriate disease tolerance might entail fulminant infection or long-term pathogen persistence. The interpretation of sepsis as collapsing tolerance and resistance responses obtains increasing support by recent experimental data, in which mTOR and AMPK, as their master regulators, play central roles: For example, mortality of septic mice could be drastically decreased after treatment with the specific mTOR inhibitor rapamycin (50) and the mTOR–HIF1alpha pathway is required for metabolic activation of trained monocytes (51). On the other hand, AICAR and metformin as agonists of AMPK have been shown to prevent sepsis in mice with similar efficacy (52, 53). Moreover, while AMPK suppresses resistance responses controlled by mTOR (54, 55), mTOR blocks disease tolerance and maintenance responses mediated by AMPK (56). Reduced mortality of septic mice by treatment with either mTOR inhibitors or AMPK activating agents could thus be interpreted as either directly or indirectly suppressing excessive resistance reactions promoted by mTOR. However, recent studies report opposed effects. Under certain conditions, stimulation of AMPK by metformin significantly increases the mortality of septic mice (57), indicating that the effects are context- and dose-dependent. Besides this paradigmatic antagonism of AMPK and mTOR-related pathways, Akt and forkhead box O (FOXO) have been recently found to mediate infection-induced cachexia, as indicated by *Drosophila* FOXO mutants surviving infection longer than wt conspecifics (58). As outlined in Figure 2, we assume that dysregulated mTOR-, AMPK-, and related signaling pathways are the molecular basis underlying infection-triggered organ dysfunction. The intricate connection of metabolism, resistance responses, and disease tolerance suggests a mechanism for controlled resource allocation to either immunity or repair processes. Aggravating factors, such as comorbidities or environmental stresses, contribute to pushing disease tolerance and resistance out of this delicate equilibrium.

## Outlook—Purposeful Manipulation of Signals Mediating Damage and Repair

The recognition of the close interrelation between metabolism and host defense mechanisms opens new perspectives to sepsis treatment. Most notably, stimulating disease tolerance during sepsis might be a strategy to support patients in surviving septic shock. The fundamental concept of the hormetic nature of mild-to-moderate stress has been observed as a highly conserved phenomenon across kingdoms. Applying such principles, e.g., induction of the DNA-damage response with low doses of anthracyclines, might add to the therapeutic options in treating septic organ failure. However, timing and targeting of such interventions will be challenging. Manipulation of the relevant signals is likely to produce cell- and tissue-specific (side) effects. Hence, before signaling mediators can be used therapeutically, it will be necessary to overcome the significant hurdles posed by these multifunctional signaling molecules. Nanoparticle-based targeted drug-delivery (21, 22, 59) might help to specifically tackle this problem.

The manipulation of key signaling pathways can be expected to significantly add to armamentarium of sepsis therapy, in particular, in the light of increasing antibiotic resistance.

## Author Contributions

MB, SC, ML, SW, BL, and RW wrote sections of the manuscript. All authors contributed to manuscript revision, read and approved the submitted version.

## Conflict of Interest Statement

MB declares that he is co-founder and share-holder of a company aiming to develop PI3K inhibitors for target-specific treatment of organ dysfunction. RW holds patents related to the therapeutic use of PI3K inhibitors. All other authors declare that the research was conducted in the absence of any commercial or financial relationships that could be construed as a potential conflict of interest.

## References

[1] Singer M Deutschman CS Seymour CW Shankar-Hari M Annane D Bauer M The third international consensus definitions for sepsis and septic shock (Sepsis-3). JAMA (2016) 315:801–10. 10.1001/jama.2016.0287 26903338 PMC4968574

[2] Bone RC. Toward an epidemiology and natural history of SIRS (systemic inflammatory response syndrome). JAMA (1992) 268:3452–5. 10.1001/jama.268.24.3452 1460735

[3] Levy MM Fink MP Marshall JC Abraham E Angus D Cook D 2001 SCCM/ESICM/ACCP/ATS/SIS international sepsis definitions conference. Intensive Care Med (2003) 29:530–8. 10.1007/s00134-003-1662-x 12664219

[4] Medzhitov R Schneider DS Soares MP. Disease tolerance as a defense strategy. Science (2012) 335:936–41. 10.1126/science.1214935 22363001 PMC3564547

[5] Weber M Lambeck S Ding N Henken S Kohl M Deigner HP Hepatic induction of cholesterol biosynthesis reflects a remote adaptive response to pneumococcal pneumonia. FASEB J (2012) 26:2424–36. 10.1096/fj.11-191957 22415311

[6] Singer M. The role of mitochondrial dysfunction in sepsis-induced multi-organ failure. Virulence (2014) 5:66–72. 10.4161/viru.26907 24185508 PMC3916385

[7] Weis S Carlos AR Moita MR Singh S Blankenhaus B Cardoso S Metabolic adaptation establishes disease tolerance to sepsis. Cell (2017) 169:1263–1275.e14. 10.1016/j.cell.2017.05.031 28622511 PMC5480394

[8] Merx MW Weber C Sepsis and the heart. Circulation (2007) 116:793–802. 10.1161/CIRCULATIONAHA.106.678359 17698745

[9] Thomas-Rueddel DO Poidinger B Weiss M Bach F Dey K Häberle H Hyperlactatemia is an independent predictor of mortality and denotes distinct subtypes of severe sepsis and septic shock. J Crit Care (2015) 30:439.e1–6. 10.1016/j.jcrc.2014.10.027 25466313

[10] Opal SM van der Poll T. Endothelial barrier dysfunction in septic shock. J Intern Med (2015) 277:277–93. 10.1111/joim.12331 25418337

[11] Carré JE Orban J-C Re L Felsmann K Iffert W Bauer M Survival in critical illness is associated with early activation of mitochondrial biogenesis. Am J Respir Crit Care Med (2010) 182:745–51. 10.1164/rccm.201003-0326OC 20538956 PMC2949402

[12] Reiling JH Sabatini DM. Stress and mTORture signaling. Oncogene (2006) 25:6373–83. 10.1038/sj.onc.1209889 17041623

[13] Cheng SC Scicluna BP Arts RJW Gresnigt MS Lachmandas E Giamarellos-Bourboulis EJ Broad defects in the energy metabolism of leukocytes underlie immunoparalysis in sepsis. Nat Immunol (2016) 17:406–13. 10.1038/ni.3398 26950237

[14] Whitehead MW Hainsworth I Kingham JGC. The causes of obvious jaundice in South West Wales: perceptions versus reality. Gut (2001) 48:409–13. 10.1136/gut.48.3.409 11171834 PMC1760136

[15] Hotchkiss RS Swanson PE Freeman BD Tinsley KW Cobb JP Matuschak GM Apoptotic cell death in patients with sepsis, shock, and multiple organ dysfunction. Crit Care Med (1999) 27:1230–51. 10.1097/00003246-199907000-00002 10446814

[16] Recknagel P Gonnert FA Westermann M Lambeck S Lupp A Rudiger A Liver dysfunction and phosphatidylinositol-3-kinase signalling in early sepsis: experimental studies in rodent models of peritonitis. PLoS Med (2012) 9:e1001338. 10.1371/journal.pmed.1001338 23152722 PMC3496669

[17] Bauer M Press AT Trauner M. The liver in sepsis: patterns of response and injury. Curr Opin Crit Care (2013) 19:123–7. 10.1097/MCC.0b013e32835eba6d 23448974

[18] Riedemann NC Guo RF Ward PA Novel strategies for the treatment of sepsis. Nat Med (2003) 9:517–24. 10.1038/nm0503-517 12724763

[19] Wrann CD Tabriz NA Barkhausen T Klos A Van Griensven M Pape HC The phosphatidylinositol 3-kinase signaling pathway exerts protective effects during sepsis by controlling C5a-mediated activation of innate immune functions. J Immunol (2007) 178:5940–8. 10.4049/jimmunol.178.9.5940 17442978

[20] Coldewey SM Benetti E Collino M Pfeilschifter J Sponholz C Bauer M Elevation of serum sphingosine-1-phosphate attenuates impaired cardiac function in experimental sepsis. Sci Rep (2016) 6:27594. 10.1038/srep27594 27277195 PMC4899780

[21] Press AT Traeger A Pietsch C Mosig A Wagner M Clemens MG Cell type-specific delivery of short interfering RNAs by dye-functionalised theranostic nanoparticles. Nat Commun (2014) 5:5565. 10.1038/ncomms6565 25470305 PMC4268698

[22] Press AT Ramoji A Vd Lühe M Rinkenauer AC Hoff J Butans M Cargo–carrier interactions significantly contribute to micellar conformation and biodistribution. NPG Asia Mater (2017) 9:e444 10.1038/am.2017.161

[23] Taylor NJ Nishtala A Manakkat Vijay GK Abeles RD Auzinger G Bernal W Circulating neutrophil dysfunction in acute liver failure. Hepatology (2013) 57:1142–52. 10.1002/hep.26102 23079896

[24] van Hoek AJ Andrews N Waight PA Stowe J Gates P George R The effect of underlying clinical conditions on the risk of developing invasive pneumococcal disease in England. J Infect (2012) 65:17–24. 10.1016/j.jinf.2012.02.017 22394683

[25] Bruns T Zimmermann HW Stallmach A. Risk factors and outcome of bacterial infections in cirrhosis. World J Gastroenterol (2014) 20:2542–54. 10.3748/wjg.v20.i10.2542 24627590 PMC3949263

[26] Horn P Metzing UB Steidl R Romeike B Rauchfuss F Sponholz C Chemerin in peritoneal sepsis and its associations with glucose metabolism and prognosis: a translational cross-sectional study. Crit Care (2016) 20:39. 10.1186/s13054-016-1209-5 26873079 PMC4751629

[27] von Loeffelholz C Docke S Lock JF Lieske S Horn P Kriebel J Increased lipogenesis in spite of upregulated hepatic 5’AMP-activated protein kinase in human non-alcoholic fatty liver. Hepatol Res (2017) 47:890–901. 10.1111/hepr.12825 27689765

[28] Liu SF Malik AB. NF-kappa B activation as a pathological mechanism of septic shock and inflammation. Am J Physiol Lung Cell Mol Physiol (2006) 290:L622–45. 10.1152/ajplung.00477.2005 16531564

[29] Kortgen A Paxian M Werth M Recknagel P Rauchfuss F Lupp A Prospective assessment of hepatic function and mechanisms of dysfunction in the critically ill. Shock (2009) 32:358–65. 10.1097/SHK.0b013e31819d8204 19197231

[30] Råberg L Sim D Read AF. Disentangling genetic variation for resistance and tolerance to infectious diseases in animals. Science (2007) 318:812–4. 10.1126/science.1148526 17975068

[31] Schneider DS Ayres JS. Two ways to survive infection: what resistance and tolerance can teach us about treating infectious diseases. Nat Rev Immunol (2008) 8:889–95. 10.1038/nri2432 18927577 PMC4368196

[32] Soares MP Gozzelino R Weis S. Tissue damage control in disease tolerance. Trends Immunol (2014) 35:483–94. 10.1016/j.it.2014.08.001 25182198

[33] Kotas ME Medzhitov R. Homeostasis, inflammation, and disease susceptibility. Cell (2015) 160:816–27. 10.1016/j.cell.2015.02.010 25723161 PMC4369762

[34] Ayres JS Schneider DS. Tolerance of infections. Annu Rev Immunol (2012) 30:271–94. 10.1146/annurev-immunol-020711-075030 22224770

[35] Ayres JS Freitag N Schneider DS. Identification of *Drosophila* mutants altering defense of and endurance to *Listeria monocytogenes* infection. Genetics (2008) 178:1807–15. 10.1534/genetics.107.083782 18245331 PMC2278058

[36] Howick VM Lazzaro BP. Genotype and diet shape resistance and tolerance across distinct phases of bacterial infection. BMC Evol Biol (2014) 14:56. 10.1186/1471-2148-14-56 24655914 PMC3997931

[37] Figueiredo N Chora A Raquel H Pejanovic N Pereira P Hartleben B Anthracyclines induce DNA damage response-mediated protection against severe sepsis. Immunity (2013) 39:874–84. 10.1016/j.immuni.2013.08.039 24184056 PMC3968948

[38] Ermolaeva MA Segref A Dakhovnik A Ou HL Schneider JI Utermohlen O DNA damage in germ cells induces an innate immune response that triggers systemic stress resistance. Nature (2013) 501:416–20. 10.1038/nature12452 23975097 PMC4120807

[39] Weis S Rubio I Ludwig K Weigel C Jentho E Hormesis and defense of infectious disease. Int J Mol Sci (2017) 18:1273 10.3390/ijms18061273 PMC5486095 28617331

[40] Fauci AS Morens DM The perpetual challenge of infectious diseases. N Engl J Med (2012) 366:454–61. 10.1056/NEJMra1108296 22296079

[41] Aminov R. History of antimicrobial drug discovery: major classes and health impact. Biochem Pharmacol (2017) 133:4–19. 10.1016/j.bcp.2016.10.001 27720719

[42] Mayr FB Yende S Angus DC. Epidemiology of severe sepsis. Virulence (2014) 5:4–11. 10.4161/viru.27372 24335434 PMC3916382

[43] Medzhitov R. Septic shock: on the importance of being tolerant. Immunity (2013) 39:799–800. 10.1016/j.immuni.2013.10.012 24238335

[44] Schafer JF Tolerance to plant disease. Annu Rev Phytopathol (1971) 9:235–52. 10.1146/annurev.py.09.090171.001315

[45] Larsen R Gozzelino R Jeney V Tokaji L Bozza FA Japiassu AM A central role for free heme in the pathogenesis of severe sepsis. Sci Transl Med (2010) 2:51ra71. 10.1126/scitranslmed.3001118 20881280

[46] Sahoo M Del Barrio L Miller MA Re F. Neutrophil elastase causes tissue damage that decreases host tolerance to lung infection with *Burkholderia* species. PLoS Pathog (2014) 10:e1004327. 10.1371/journal.ppat.1004327 25166912 PMC4148436

[47] Miller MR White A Boots M. The evolution of parasites in response to tolerance in their hosts: the good, the bad, and apparent commensalism. Evolution (2006) 60:945–56. 10.1554/05-654.1 16817535

[48] Kahl BC Becker K Loffler B. Clinical significance and pathogenesis of staphylococcal small colony variants in persistent infections. Clin Microbiol Rev (2016) 29:401–27. 10.1128/CMR.00069-15 26960941 PMC4786882

[49] Wood TK. Combatting bacterial persister cells. Biotechnol Bioeng (2016) 113:476–83. 10.1002/bit.25721 26264116

[50] Lee PS Wilhelmson ASK Hubner AP Reynolds SB Gallacchi DA Chiou TT mTORC1-S6K activation by endotoxin contributes to cytokine up-regulation and early lethality in animals. PLoS One (2010) 5:e14399. 10.1371/journal.pone.0014399 21200439 PMC3006197

[51] Cheng S-C Quintin J Cramer RA Shepardson KM Saeed S Kumar V mTOR- and HIF-1 alpha-mediated aerobic glycolysis as metabolic basis for trained immunity. Science (2014) 345:1250684 10.1126/science.1250684 25258083 PMC4226238

[52] Escobar DA Botero-Quintero AM Kautza BC Luciano J Loughran P Darwiche S Adenosine monophosphate-activated protein kinase activation protects against sepsis-induced organ injury and inflammation. J Surg Res (2015) 194:262–72. 10.1016/j.jss.2014.10.009 25456115 PMC4346495

[53] Mulchandani N Yang WL Khan MM Zhang F Marambaud P Nicastro J Stimulation of brain AMP-activated protein kinase attenuates inflammation and acute lung injury in sepsis. Mol Med (2015) 21:637–44. 10.2119/molmed.2015.00179 26252187 PMC4656202

[54] Gwinn DM Shackelford DB Egan DF Mihaylova MM Mery A Vasquez DS AMPK phosphorylation of raptor mediates a metabolic checkpoint. Mol Cell (2008) 30:214–26. 10.1016/j.molcel.2008.03.003 18439900 PMC2674027

[55] Rutherford C Speirs C Williams JJL Ewart MA Mancini SJ Hawley SA Phosphorylation of Janus kinase 1 (JAK1) by AMP-activated protein kinase (AMPK) links energy sensing to anti-inflammatory signaling. Sci Signal (2016) 9:10. 10.1126/scisignal.aaf8566 27919027

[56] Hardie DG. AMP-activated protein kinase: a key regulator of energy balance with many roles in human disease. J Intern Med (2014) 276:543–59. 10.1111/joim.12268 24824502 PMC5705060

[57] Zha QB Wei HX Li CG Liang YD Xu LH Bai WJ ATP-induced inflammasome activation and pyroptosis is regulated by AMP-activated protein kinase in macrophages. Front Immunol (2016) 7:597. 10.3389/fimmu.2016.00597 28018360 PMC5149551

[58] Dionne MS Pham LN Shirasu-Hiza M Schneider DS. Akt and foxo dysregulation contribute to infection-induced wasting in *Drosophila*. Curr Biol (2006) 16:1977–85. 10.1016/j.cub.2006.08.052 17055976

[59] Fitzgerald K White S Borodovsky A Bettencourt BR Strahs A Clausen V A highly durable RNAi therapeutic inhibitor of PCSK9. N Engl J Med (2017) 376:41–51. 10.1056/NEJMoa1609243 27959715 PMC5778873

